# Optimisation of Garbage Bin Layout in Rural Infrastructure for Promoting the Renovation of Rural Human Settlements: Case Study of Yuding Village in China

**DOI:** 10.3390/ijerph182111633

**Published:** 2021-11-05

**Authors:** Yue Shen, Cheng Wang

**Affiliations:** School of Geographical Sciences, Southwest University, Chongqing 400715, China; cactus_sy@163.com

**Keywords:** rural human settlements, rural infrastructure, garbage bins, ANT, layout optimisation

## Abstract

Optimising the layout of garbage bins is a requirement for improving the utilisation efficiency of rural infrastructure and continuously promoting the renovation of rural human settlements in China. This study selects Yuding Village in Chongqing, China, as the study area. The present distribution of garbage bins and the existing problems are analysed on the basis of interview materials and the point on interest data of garbage bins obtained from an on-the-spot investigation. Actor network theory (ANT) is suitable for small-scale micro research, and thus, this study uses ANT to construct a research framework for garbage bin layout optimisation. Then, it designs an optimisation path for the layout of garbage bins in Yuding Village by identifying different actors and their common interests, classifying the transformation of roles amongst various actors and building a stable heterogeneous network that can be used as a guide for determining the optimal spatial layout of garbage bins. This study combines a sociological theory with geospatial phenomena, providing a new idea for studying the optimal layout of infrastructure.

## 1. Introduction

The United Nations (UN) Conference on Human Settlements, which is committed to the improvement of the quality of human settlements, has been successfully held three times from the 1970s to the beginning of the 21st century [1,2]. The UN Human Settlements Programme emphasizes the realisation of the sustainable development of human settlements at different levels, namely, cities, towns and villages [3,4,5]; however, the quality of human settlements in rural areas requires more concern in accordance with the principle of fairness of sustainable development. As a traditional agricultural country and following the objective of the UN, the government of China has given considerable attention to issues regarding rural human settlements [6]. In his speech delivered during the 19th National Congress of the Communist Party of China on 18 October 2017, President Xi Jinping emphasised solving prominent environmental problems, taking measures to improve rural human settlements and improving the treatment of solid waste and garbage [7]. In February 2018, the Chinese central authorities released the “Three-year Action Plan for the Renovation of Rural Human Settlements”. The treatment of solid waste is regarded as one of the key tasks of this plan. In accordance with the three-year action plan, a collection–transportation–disposal system of domestic waste that is in line with the reality of rural areas in various ways should be established to promote the treatment efficiency of rural domestic waste. During the annual central rural work conference held in Beijing on 28 December 2020, President Xi pointed out that the focus of work should be shifted to the overall revitalisation of rural areas, focus on the construction of public infrastructure in rural areas should be continued and the improvement and upgrading of rural human settlements should be promoted. 

Rural infrastructure is an important part of the hard environment of human settlements [8]; it plays an essential role in improving rural livelihoods and enhancing sustainable and environmentally friendly agricultural production [9]. While as a type of rural living infrastructure funded by the government [10], the utilisation efficiency of garbage bins is directly related to the quality of rural human settlements. For developing countries like China, rural infrastructure is crucial for rural development [11]. In line with the implementation of a coordinated urban–rural development strategy, infrastructure investment in China has been intentionally shifted to rural areas [12]. Even with the constantly increase of financial expenditure, the rural solid waste problems in some areas are still prominent [13], which can assuredly cause serious environmental pollution. The relationship between environment and health is of extreme relevance in public health [14], once domestic waste is not disposed of properly, it poses a threat to public health [15]. Locating waste collection bins sit is a portion of solid waste management (SWM) [16], and is of great significance to improve the work efficiency of SWM.

Some excellent progress has been made in the related research on the optimal layout of garbage bins worldwide. SWM as a main challenge for the local government to optimise the financial budget [17], scholars often measure the quantity and location of garbage bins from a cost–benefit point of view [18,19], and attempt to find the optimal state of facility layout in such way. In addition, the garbage bin layout optimalisation is always summed up as a geospatial optimisation problem [20], thus, geographical analysis is compulsory to reveal the local disparities [21]. Some spatial relationship between quantity and location of them has also been discussed through the mathematical model or formulation [22,23]. Additionally, in some local practise, multi-objective programming approach to tackle the location-allocation problem of waste management facilities [24]. The optimal layout of garbage bins is a comprehensive and social issue, which involves the realization of multiple subject goals, so more elements should be taken into consideration. 

Most studies consider the realisation of a single main goal (e.g., the effectiveness of local government investment; the improvement of environmental pollution or the convenience of residents’ daily life) while lack of comprehensive embodiment. In the existing studies, the statistical relationships among phenomena were generally discussed, but operational processes and reasons among them were ignored. The optimal layout of garbage bins considered in this study emphasizes the realisation of the interests of multiple behaviour subjects on a micro spatial scale (a village scale). Therefore, we attempted to use a scientific theory as guideline, and construct a research analysis framework based on this, hoping to guide the optimal layout process of village garbage bins through this framework.

## 2. Research Analysis Framework Based on ANT

Actor network theory (ANT) derived from sociology has been proven to be applicable to solving the type of problem that refers to geospatial optimisation problem that refers to multiple behaviour subjects at the micro level. ANT is one of the representatives in the sociology of scientific knowledge; it focuses on three concepts: actor, heterogeneous network and translation [25]. ANT is about the process of constructing and maintaining heterogeneous networks by human or nonhuman actors via the process of translation. Actor (actant/agent) is the most basic concept in ANT. It includes not only humans but also different types of nonhumans, including material things, immaterial things (e.g., concepts and morals) and actions [26]. ANT advocates the “general symmetry principle”, and it holds that the interpretation function of nature and society in scientific knowledge should be viewed symmetrically. On the basis of this principle, Latour believed that human and nonhuman elements in nature and society should be treated equally [27]. An equal view of the roles of humans and nonhumans in a certain social phenomenon is a dominant sign that ANT differs from other sociological theories.

Although this theory originated from sociology, it has been discussed in various academic fields [28]. On the basis of ANT’s symmetrical view of human and nonhuman actors and the research model for considering the interactions among actors through network relations, this theory cannot only strengthen the theoretical construction of different fields and phenomena but can also provide an effective analysis method for spatial relations and policy practice research in the fields of rural and urban studies, economics and tourism [29,30,31,32]. Additionally, ANT has unique understanding of space, so it also has been widely used in the study of rural geography, including the construction of agricultural space [33], rural development [34], supply of agricultural products [35], distance in the process of rural economic development [36] and spatial transformation of traditional villages [37]. From the structural perspective, ANT constructs the relationship between actors through the flow of elements and the form of grid interaction, which can be applied to research on microcosmic material space [38].

**Figure 1:**
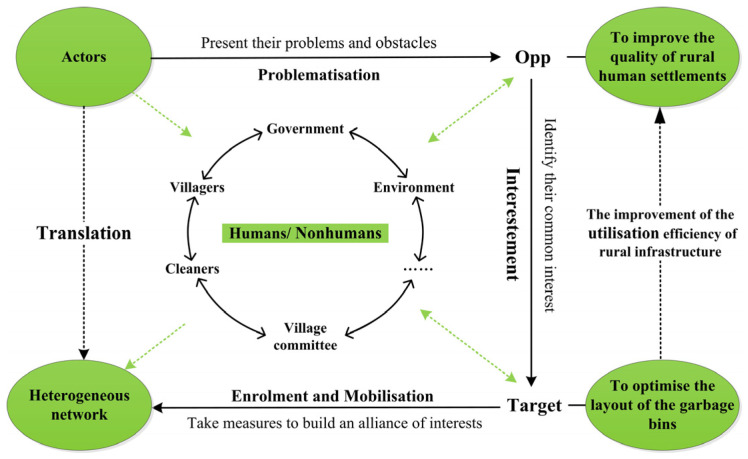
Research framework for garbage bin layout optimisation based on ANT.

Applying the theory of ANT, a framework will be constructed to handle with the optimisation of the spatial layout of garbage bins (Figure 1). The first part of the research framework refers to the actors. The optimal layout of rural garbage bins involves not only the optimisation of the spatial layout of geography but also the balance amongst the government, villagers, cleaners and the environment. The process of translation, which includes four stages, namely, “Problematisation”, “Interestement”, “Enrolment” and “Mobilisation”, can form connections among different actors and is the key to constructing a heterogeneous network. During the process of building a heterogeneous network, different actors (humans or nonhumans) will experience the transformation of identity and responsibility through translation. The translation process of optimising the layout of garbage bins can be divided into three steps.

Step 1: Problematisation (presentation of the problems and obstacles of different actors). Different actors have varying needs in the optimal layout of garbage bins. To make all the actors accept the setting of the problem, “obligatory points of passage” (OPPs) should be determined to provide the premise for the benefit-conferring process. Different actors present their problems and obstacles through “problematisation”, and OPPs are identified.

Step 2: Interestement (identification of the common interest of different actors). When actors want to achieve some of their goals, they must form alliances with other actors by finding and getting through OPPs. Actors identify their common interests through “interestement”, and then a common target, which is the key to building an alliance of interests, can be set.

Step 3: Enrolment and mobilisation (taking measures to build an alliance interests). The core actor and the proponent should be further identified during this stage. The core actor typically plays the role of an organiser in the process of optimising the layout of garbage bins, managing other actors and participating in actions through devices and strategies. Meanwhile, the proponent plays the role of “spokesperson” of the entire network alliance during “mobilisation” and exercises power over other members of the network to maintain the stability of the heterogeneous network.

The construction of the network is the process of optimising the layout of garbage bins, and the formation of heterogeneous networks helps improve the quality of human settlements. Though the developed framework, the theory can be better applied to practice, realising the optimisation of the allocation of rural infrastructure and the improvement of quality of human settlements.

## 3. Data Collection and Organisation

### 3.1. Study Area

**Figure 2:**
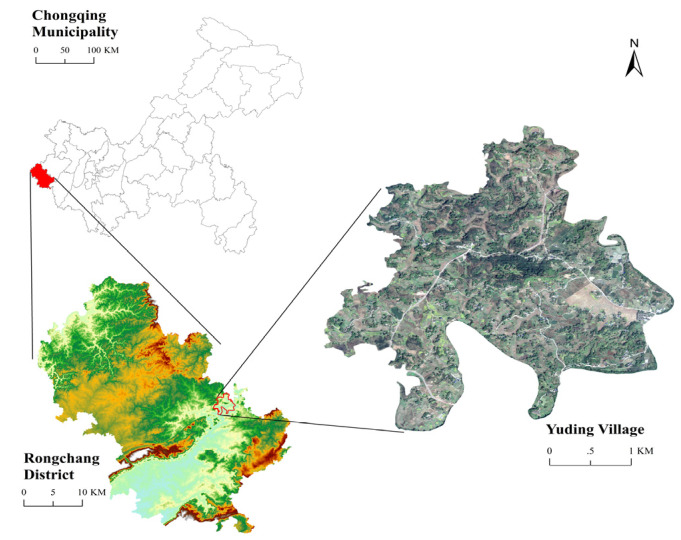
Location of Yuding Village.

Yuding Village, located in Wanling Town, Rongchang District, Chongqing Municipality, China, is located between 29°27′15″–29°27′58″ E and 105°36′32″–105°37′21″ N (Figure 2). It covers an area of 760.81 hm^2^ (agricultural space: 512.61 hm^2^; ecological space: 167.42 hm^2^; construction space: 80.78 hm^2^). The total number of households is 1030, the residential population in the village is approximately 3000 and the residents mostly plant rice, fruits and vegetables. In 2018, the municipal government of Chongqing issued the “Chongqing Three-year Action Plan for the Renovation of Rural Human Living Environment (2018–2020)”. Districts and counties responded positively and accelerated the renovation process of rural human settlements. In 2019, Rongchang District selected 10 exemplary villages for renovating rural human settlements. Yuding Village was amongst the 10. In July 2020, the Chongqing Agriculture and Rural Affairs Commission issued “the list of the first batch of 458 beautiful liveable villages in Chongqing”, and the list includes Yuding Village. For the treatment of domestic waste in the renovation of the living environment, the model of “Village Collection, Town Transfer, County Treatment” has been adopted by the authorities. Through this model, the collection and transportation system of rural domestic waste has been fully covered, and the population coverage rate of the effective treatment of rural domestic waste has reached 100%.

### 3.2. Data Collection and Processing

**Figure 3:**
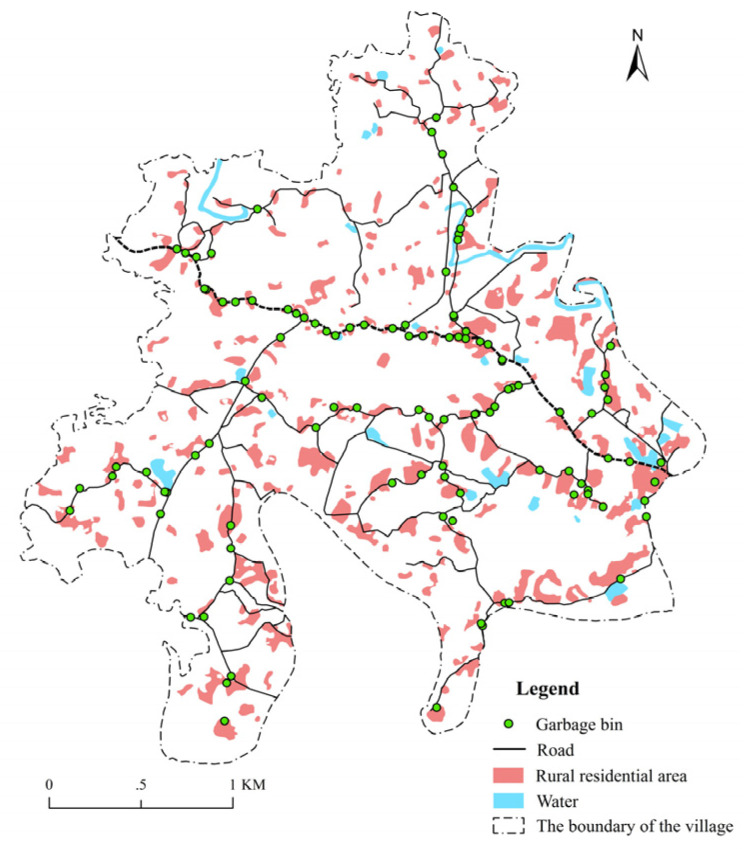
Distribution of garbage bins in Yuding Village.

We conducted an on-the-spot investigation in Yuding Village during 12 to 17 August 2019. The investigation was divided into two major stages. In the first stage, different actors were interviewed. They were selected typical representatives of different groups, including the Wanling town government, Yuding village committee and Yuding villagers and cleaners, with a total number of 26, to understand the role they played in the layout of garbage bins in the village and the targets they want to achieve (Table 1). A semi-structured interview method was used in our investigation, and the purpose of our investigation was explained to all interviewees, after which consent was received from each interviewee. The second stage involves the acquisition of the number and location information of garbage bins through a village-wide investigation. The point of interest (POI) is a type of point-like vector data that represents an actual geographical entity, and it can reflect the space and attribute information of geographic entities [39]. First-hand POI data obtained through an on-the-spot investigation exhibit the advantages of strong credibility, high accuracy and ample content. Through our on-the-spot investigation in Yuding Village, and with the help of “2bulu” (Shenzhen 2bulu Information Technology Co., Ltd., Shenzhen, China) (a mobile phone auxiliary application for data acquisition and storage and trajectory recording, tracking and positioning), we regarded each garbage bin as a POI and added its location information to the high-definition remote sensing data of the mobile phone application. After two days of data collection, 107 POIs of garbage bins in Yuding Village were obtained. To ensure the timeliness of the data sources used in this study, we went back to Yuding Village for 1 day in March 2021 to conduct a re-investigation. Through data sampling verification, the layout of garbage bins in Yuding Village in 2021 and 2019 was determined to exhibit a high degree of coincidence. Therefore, this study adopted the 107 POIs obtained in 2019 as the data source for further research. In addition, we also visited the Rongchang District Planning and Natural Resources Bureau to obtain the vector data of the administrative boundary, rural residential areas and roads in Yuding Village. To facilitate the follow-up study, we exported the stored POI format in “2bulu” into Keyhole Markup Language (KML) and then convert the data format from KML into vector data through ArcGIS. The spatial distribution of garbage cans in Yuding Village and their relative position relationship with other geographical entities can be obtained by superimposing the vector data with the POIs obtained in the on-the-spot investigation through ArcGIS (Figure 3). 

**Figure 4:**
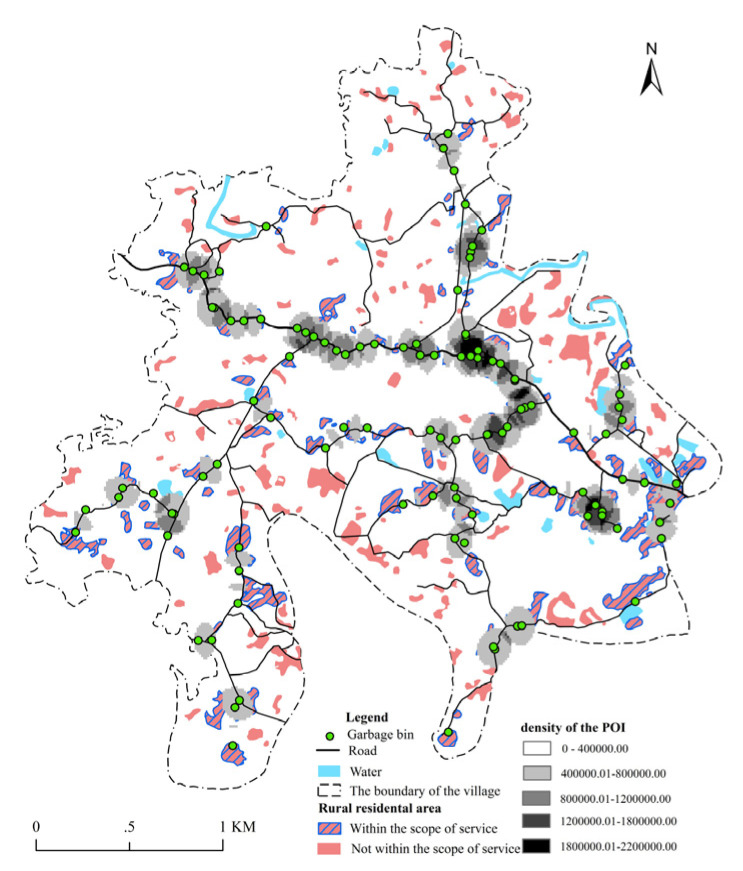
Spatial layout rationality of garbage bins in Yuding Village.

Moreover, with the help of the spatial analysis tool of ArcGIS and setting the 107 POIs obtained from the on-the-spot investigation as the object, the point density was determined, buffer analysis was conducted (the standard service radius of municipal solid waste collection points is not more than 100 m in accordance with the relevant requirements for village planning), the relative position relationship between the garbage bins and residential areas and roads in the village was explored and the spatial distribution pattern and service scope of garbage bins in Yuding Village were analysed (Figure 4).

## 4. Results

### 4.1. Problematisation Processes Derived from Different Actors

#### 4.1.1. Town Government

The town government, which has the duty and obligation to organise the preparation of village planning and the allocation of rural public resources, is the grassroots administrative organ of a country that is responsible for the direct management of a village. The town government is in charge of the realistic implementation of the central government’s policies and the provision of a multitude of services [40]. As early as 2012, the Wanling town government developed a special environmental protection plan and invested a considerable amount of financial funds in garbage removal and transportation to buy garbage bins. Upon interviewing the government officials, we learned that Wanling Town has established a complete domestic waste collection and transportation system that applies the concept of “household collection, town transportation and district disposal”. In the renovation plan for rural human settlements in Wanling Town presented in 2019, the town government planned to distribute 2400 garbage bins and improved the rural infrastructure. However, we found during the on-the-spot investigation that most of these garbage bins are distributed in built-up areas and the number of garbage bins in the countryside is relatively small. To facilitate transportation, these garbage bins are distributed on major roads within the village. In general, the principle adopted in allocating public resources is to pursue efficiency first. When relevant staff members of the town government have insufficient knowledge about the village, the government’s allocation of public resources may be frequently inconsistent with supply and demand, and thus, cannot fully meet the needs of rural residents. In the current study, the government of Wanling Town holds the leading power of the problem of garbage bin configuration and layout. It has committed to purchase garbage bins and supervise the village committees of Yuding for their delivery and placement. The excessive pursuit of efficiency, disregard for the principle of fairness and ineffective supervision can easily lead to confusion in the layout of garbage bins, and thus, they cannot fully play their role in serving the daily life of rural residents.

#### 4.1.2. Village Committee

The village committee is a form of villagers’ autonomous organisation. It is considered a mass organisation of self-government, farmers’ autonomous self-management, self-education and self-service [41]. Although the village committee is considerably more familiar with the relevant situation of Yuding Village than the government of Wanling Town, the management and maintenance of garbage bins have not been given attention to in village affairs. During the on-the-spot investigation in Yuding Village, a lack of management and maintenance was found after the configuration of public resources. This situation may lead to the contradiction in which the service radius of the garbage bins overlaps and most villagers cannot enjoy the convenience of life brought by garbage bins. The village committee is the most basic organisation that manages human and nonhuman elements (e.g., villagers and garbage bins, respectively) and promotes the coordination of supply and demand between them. The effectiveness of the behaviour of a management subject determines the effectiveness of management to a large extent. Therefore, the specific responsibilities of the staff of the village committee must be clarified to include the renovation of rural human settlements and the management and maintenance of rural infrastructure within the scope of their daily work.

#### 4.1.3. Villagers

The villagers of Yuding Village are the direct beneficiaries of public resources, and they provide the most intuitive perception of whether the layout of garbage bins is reasonable. This intuitive perception can be measured by the number and location of garbage bins. The daily output of domestic waste per person in Yuding Village is estimated to be approximately 0.8 kg. In accordance with the calculation of the 20 kg load-bearing capacity of a single garbage bin, the number of garbage bins needed in Yuding Village should not be less than 120. From the perspective of location, the distribution of garbage bins should be highly consistent with that of rural residential areas to facilitate the daily life of villagers. However, through spatial analysis we found that the shortest straight-line distance between two adjacent data points is only 10 m, whereas the longest is more than 600 m. The spatial distribution of garbage bins in Yuding Village is uneven, which is a non-normal distribution in rural residential areas. A spatial overlap occurs in the service scope of a single garbage bin, and the area of the rural residential within the buffer zone was further calculated. Only 43.19% of the entire rural residential area is within the service radius of the garbage bins, whereas the residents of the remaining more than 50% do not enjoy the convenience of life brought by the garbage bins. In the interview, the local villagers who live far from the garbage bins believe that the distribution of garbage bins in the village is unreasonable, and thus, they hold the attitude that the bins should be rearranged. Villagers are the service objects of public resources; the town government allocates public resources and the village committee manages public resources. When planning the layout of garbage bins, the convenience of the villagers’ daily life should be set as an important goal, the principle of fairness should be considered and all residents in the village must enjoy the convenience brought by public service resources. Only in this manner can the government provide the material basis for improving the living environment to the greatest extent.

#### 4.1.4. Cleaners

The duty of the cleaners is to ensure the cleanliness of public areas in the village, including cleaning and transporting domestic garbage produced by the villagers. At present, Yuding Village has only one full-time and four part-time cleaners. From our interview with the cleaners, we determine that the service range of cleaners refers to the spatial distribution of garbage bins. The high consistency between the location of garbage bins and the distribution of roads in the village can reduce the workload of cleaners, facilitate garbage cleaning and transportation to the greatest extent and improve the work efficiency of cleaners. When point density analysis results were superimposed with the data of rural residential areas and roads in Yuding Village, the high-density distribution area was found to be concentrated at the centre of the village, and the spatial distribution of high-density areas is consistent with the road distribution. Moreover, the distribution density of garbage bins on both sides of the village’s major roads is evidently higher than that in areas without road distribution. The distribution density of garbage cans in the south and north of the village is significantly lower than that at the centre of the village. In terms of service range, the service range of the garbage bins is highly consistent with the segment range of the road. This finding agrees well with the results of the point density analysis. The current layout of garbage bins in Yuding Village can facilitate the cleaner’s daily work to a certain extent.

#### 4.1.5. Environment

The typical nonhuman actors constructed by the actor network in the optimal layout of garbage bins are highly responsive to the behaviour of human actors. To a certain extent, the quality of rural human settlements can reflect the stability of human actors, such as governments, village committees, villagers and cleaners, in building heterogeneous networks. However, the environment can provide feedback to a series of actors’ behaviour, establishing the interaction between human and nonhuman actors. At present, one of the environmental problems faced by Chinese villages is the spread of non-point source (NPS) pollution and the resulting threat to public health [42]. The unreasonable layout of garbage bins is an important cause of NPS pollution [43].

### 4.2. Targets in Optimising the Layout of Garbage Bins

**Figure 5:**
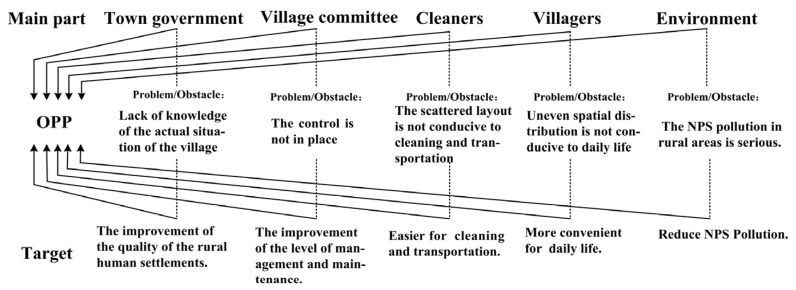
Actors and OPP in optimising the layout of garbage bins.

The ultimate purpose of “problematisation” is to find an OPP and form a heterogeneous network. Each actor exhibits different behaviour or responses to the behaviour of various subjects based on varying interests and demands. The town government is the primary body with relative rights, and its mission is to complete the task of renovating human living environment as ordered by the higher government and improve the utilisation efficiency of public resources. The village committee is an intermediate subject based on the connection between grassroots autonomy and government jurisdiction, and its mission is to enhance the effectiveness of the daily management of village affairs and promote the improvement of the village environment. Villagers are not only the subject of demands, but also the object of management. Their interest is based on their own practical needs, i.e., to facilitate their daily life and improve the quality of rural human settlements. The duty of cleaners is to ensure the cleanliness of the village space and prevent the expansion of the scope of NPS pollution. As the bearing space of human actors, the quality of the environment also results from the integration of the interests of different actors(Figure 5). Comprehensively considering the interests and demands of various actors, we can find out the balance of various interests, i.e., to build the OPP of the heterogeneous network: to improve the quality of rural human settlements in Yuding Village Different actors are linked under the guidance of the OPP by a common target, i.e., to optimise the layout of garbage bins in Yuding Village.

### 4.3. Constructing the Actor Network in Accordance with the Targets 

**Figure 6:**
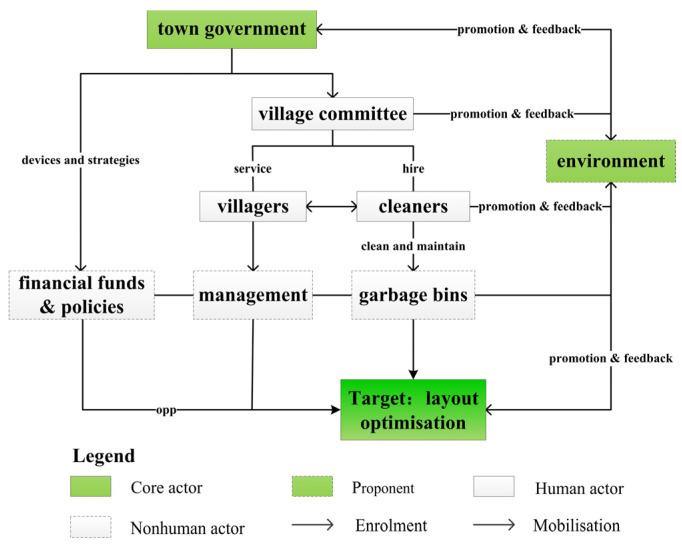
Construction of a heterogeneous network.

The construction of the actor network for the optimal layout of garbage bins in Yuding Village takes the government to the starting point, connects human actors (e.g., village committees, villagers and cleaners) and fully considers nonhuman actors (e.g., garbage bins and the environment). Funds and policies must be used and certain management practices must be adopted to determine the logical relationship between the optimal layout of garbage bins and the improvement of the quality of rural human settlements, and eventually, the ultimate goal through translation (Figure 6). The core actor and its role in network construction should be further identified through enrolment. In constructing this heterogeneous network, the town government should play the role of the core actor. Through financial funds, the town government purchases a certain number of garbage bins, which can be the material basis for improving the convenience of life of Yuding Village residents. Simultaneously, the government pays the wages of and provides basic living security to cleaners. By introducing corresponding policies (such as assigning relevant tasks to improve rural human settlements), lead different actors to give feedback jointly achieve the optimal layout of garbage bins. In completing the construction of the network alliance, another special actor should be identified, namely, the proponent. The proponent will play the role of the network’s spokesperson through mobilisation, exercise power over the network alliance and maintain its stability. The proponent is identified as the environment. As a typical nonhuman actor, the environment plays an important role in constructing a heterogeneous network. Improving the quality of rural human settlements in Yuding Village is one of the common goals pursued by all the actors in constructing an actor network for optimising the layout of garbage bins in Yuding Village. The environment responds to the behaviour of the actors driven by common interests, as reflected in the quality of human living environment in Yuding Village. An interactive relationship of promotion and feedback exists between the environment and other actors, and the level of environmental quality has become a direct force that guide other actors in formulating strategies for maintaining the stable operation of an actor network. That is, the environment has the duty of mobilisation in the construction of an actor network. It becomes the spokesperson after the construction of the actor network for the optimal layout of garbage bins in Yuding Village, and it exercises power over other members of the network alliance. Finally, the purpose of maintaining the normal operation of the network can be achieved.

### 4.4. Results of the Optimised Layout of Garbage Bins

**Figure 7:**
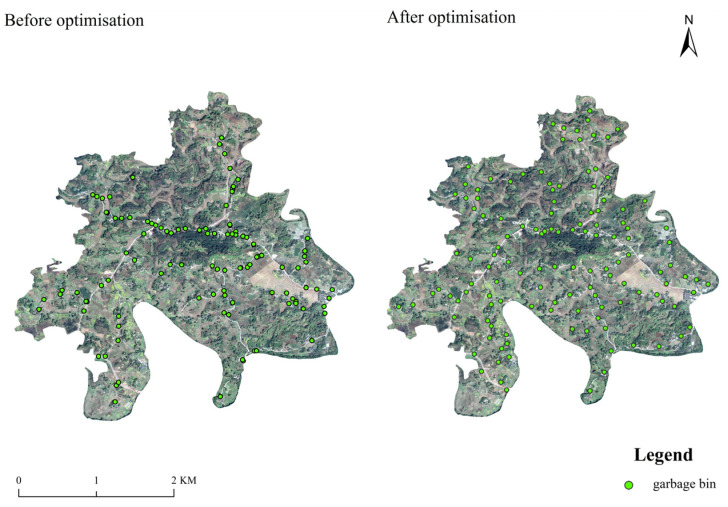
Optimised layout of garbage bins in Yuding Village.

The layout of garbage bins in Yuding Village is optimised (Figure 7) by comprehensively considering the government’s financial expenditure, the management and operation of the village committee, the convenience of villagers’ daily life, the cleaning efficiency of cleaners and the carrying capacity of the environment, with the help of the spatial analysis and visualisation functions of ArcGIS software. After optimisation, under the condition that Governmental financial expenditure increases by only CNY 5000, the number of garbage bins in Yuding Village will increase to 128, and the number of garbage bins per household will increase from 0.103 to 0.124. This slight increase makes 93.22% of rural settlements will cover the service radius of garbage bins (Table 2). The area with the densest distribution of garbage bins is near the rural trunk road in the middle of Yuding Village. From the perspectives of the town government, village committee and cleaners, the layout of garbage bins in Yuding Village must adhere to the principle of being close to the major roads within the village, which is a requirement for improving its management efficiency. For all the residents of Yuding Village, the layout of garbage bins is within the most appropriate radius of their daily activities, and the number of garbage bins in residential areas with a high population density should be increased accordingly. To effectively restrain the occurrence of NPS pollution in rural areas, the distribution of garbage bins in the entire village should be kept uniform as much as possible.

## 5. Discussion

Human settlement environment is a multilevel and multi-type spatial system that can be divided into urban and rural human settlements [44,45]. Meanwhile, under the background of China’s historical development and policy guidance in the past few decades, research results on human settlements are basically limited to the urban field [46,47,48,49]. Only a few research achievements have been achieved on the theory and practice of rural human settlements, and these achievements tend to be from macro research, such as rural planning, human-land relationship and rural transformation. A rural human settlement is a geographical space with certain social characteristic. Since the reform and opening up of China in 1978, its rural areas have been experiencing a huge transformation, and remarkable achievements have been made in agriculture, rural areas and for rural residents [50,51]. However, during rapid industrialisation and urbanisation, most rural waste and sewage discharge are poorly managed, causing severe environmental pollution [52]. Meanwhile, it also poses a great threat to public health in rural areas. With the rising income level of rural residents and under the premise that their material needs are met, more rural residents will begin to pursue spiritual satisfaction, which is primarily reflected in the need for improving the quality of the living environment. Considering that the principal contradiction of the Chinese society has been transformed into the contradiction between unbalanced and inadequate development and the people’s ever-growing needs for a better life, taking certain measures to improve the quality of rural human settlements has become imperative. 

The proposal of the Three-year Action Plan for the Renovation of Rural Living Environment provides a policy formulated for accelerating the improvement of rural human settlement environment and identifies new directions for research on the expansion of rural human settlements, namely, to provide systematic ideas for the concrete implementation of renovation and criteria for the performance evaluation of the renovation of rural human settlements. With the overall victory of the fight against poverty, China has entered into a period of promoting rural revitalisation in a multifaceted manner to achieve the goal of pleasant living environments, and thus, a five-year campaign to improve the rural living environment was launched in 2021. Starting from different renovation processes, we can also explore the implementation path of renovation from a microscopic point of view and then broaden the research scope of rural human settlements. Studying the optimal layout of village garbage bins has proposed the optimisation direction of implementing the renovation of rural human settlements from a microscopic point of view. Presenting Yuding Village as the example, we used ANT to optimise the layout of garbage bins, providing a new idea for infrastructure layout in the follow-up village planning.

The optimisation of the layout of garbage bins in rural human settlements is an optimisation problem that should consider different social relations. When implementing the layout of garbage bins in rural areas, we should consider the realisation of the interests of different actors, explore the practical needs of different stakeholders, find their common goals and build a stable social network relationship. By including the spatial behaviour of different actors in the study of renovating rural human settlement systems, the functional evolution and development trend of a macro system from a micro perspective can be explored, particularly under the background of urbanisation. Studying the optimisation path of rural human settlements from a microscopic perspective is not only conducive to the sustainable development of rural areas, but it also plays an important guiding role in the orderly development of urban and rural spaces and the healthy development of urbanisation, exhibiting certain theoretical significance and practical value.

This study identifies the human and nonhuman actors in optimising the layout of garbage bins in Yuding Village, improving the living environment of the village as the OPP, fully exploring the changes in the roles and responsibilities of various actors (translation process) and building an actor network based on the optimal layout of garbage bins in Yuding Village. We focus on the process of optimising the layout of garbage bins and constructs a heterogeneous network. However, once the construction of this network is completed, the roles and tasks of the members of the network alliance will change, and the purpose of this change is to maintain the stable operation of the heterogeneous network. However, this aspect has not included in the current study. Maintaining the stable operation of a network alliance can be regarded as one of the key points in future research. In addition, as a typical sociological theory, ANT is applied to the study of the optimal layout of rural garbage bins, defining the role played by various actors in optimising the layout and their process for building heterogeneous networks, emphasising the significance of its theoretical guidance. In addition, one limitation of this study refers to whether the interests of different actors will be realised with the optimisation of the layout of garbage bins cannot be verified by ANT. Later, the modified scheme can be applied to practice to prove that the optimised scheme is more in line with the needs of actors.

## 6. Conclusions

The treatment of domestic waste is one of the key tasks in the renovation of rural human settlements in China. Analysing the rationality of the layout of rural garbage bins and optimising their layout in accordance with the current situation cannot only improve the efficiency of the use of public resources, but also enhance the quality of life of rural residents, effectively control NPS pollution and then provide a kind of solution to improve public health problems. By analysing the spatial distribution pattern and service scope of garbage bins in Yuding Village, we determine the insufficient quantity and unequal distribution of garbage bins in this village. Adopting certain methods to research the optimal layout of garbage bins is necessary. The optimal layout space of garbage bins is a heterogeneous space that should consider the behaviour of different subjects. The optimal layout of garbage bins is not only an optimisation process of space but also a process that requires the participation of different actors. Conducting an in-depth analysis of the problem by using ANT is reasonable and necessary. Through an in-depth analysis of the three core elements of ANT, this study identifies human and nonhuman actors that play a role in the optimal layout of garbage bins in Yuding Village and defines the common interests of all the actors. The translation process is clarified, and a heterogeneous network alliance of common participants is built to achieve the objectives of optimising the layout of garbage bins and improving the quality of rural human settlements in Yuding Village.

## Figures and Tables

**Table 1:** Interviewees and key points.

Interviewees	Number	Key Points	Duration
Civil servants from Wanling Town government	3	The present situation of rural human settlements renovation, quantity and amount of garbage bins purchased, devices and strategies used in the layout of garbage bins, etc.	115 min
Staffs of village committee	2	Basic introduction of Yuding Village, population structure, current situation of management and maintenance of garbage bins, etc.	96 min
Local villagers of Yuding Village	18	The frequency and distance of littering, per capita daily garbage output, satisfaction degree with the utilization of garbage bins, etc.	362 min
Full-time cleaners in Yuding Village	3	Main work content, the area responsible for cleaning, average daily working hours, wage income, etc.	82 min

**Table 2:** Contrast items before and after optimising the layout of garbage bins.

Contrast Item	Before Optimisation	After Optimisation
Number of garbage bin	107	128
Average number of garbage bins per household	0.103	0.124
Government investment cost (CNY)	25,680	30,720
Scope of service (%)	43.19	93.22
